# Myoinositol and Selenium (MYSE) Supplementation Is Associated with Favorable Changes in Thyroid Parameters and Migraine Outcomes in Patients with Migraine and Hashimoto’s Thyroiditis: A Retrospective Cohort Study

**DOI:** 10.3390/nu18101554

**Published:** 2026-05-14

**Authors:** Cherubino Di Lorenzo, Maurizio Nordio, Fiorenzo Brongo, Francesco Casillo, Sabrina Basciani, Gabriele Sebastianelli, Mariano Serrao, Giorgio Di Lorenzo, Gianluca Coppola

**Affiliations:** 1 Department of Medico-Surgical Sciences and Biotechnologies, Sapienza University of Rome, Polo Pontino, 04100 Latina, Italy; brongo.fiorenzo@gmail.com (F.B.); francesco.casillo@uniroma1.it (F.C.); gabriele.sebastianelli@uniroma1.it (G.S.); mariano.serrao@uniroma1.it (M.S.); gianluca.coppola@uniroma1.it (G.C.); 2 The Experts Group on Inositol in Basic and Clinical Research and on PCOS (EGOI-PCOS), 00156 Rome, Italy; maurizionordio1@gmail.com; 3 Department of Experimental Medicine, Sapienza University of Rome, 00163 Rome, Italy; sabrinabasciani@yahoo.it; 4 Department of Systems Medicine, Tor Vergata University of Rome, 00133 Rome, Italy; di.lorenzo@med.uniroma2.it; 5 Istituto di Ricovero e Cura a Carattere Scientifico (I.R.C.C.S.)–Santa Lucia, 00179 Rome, Italy

**Keywords:** migraine, Hashimoto’s thyroiditis, selenium, myoinositol, thyroid-stimulating hormone, nutraceuticals

## Abstract

**Background/Objectives:** Migraine and Hashimoto’s thyroiditis (HT) are frequently comorbid, implying shared biological pathways. Selenium and myoinositol are involved in migraine pathophysiology, and their supplementation has been shown to improve thyroid function, particularly in individuals with HT. This study aimed to evaluate the impact of combined myoinositol and selenium (MYSE) supplementation on thyroid function and migraine outcomes in patients with migraine and HT. **Methods:** We conducted a retrospective study on a cohort of 163 adults with migraine comorbid with HT who received a 6-month MYSE supplementation. Thyroid parameters, namely thyrotropin (TSH), free thyroxine (fT4), and free triiodothyronine (fT3), and migraine features, namely monthly migraine days (MMDs), monthly migraine attacks (MMAs), and monthly symptomatic drug use (MSDs), were assessed at baseline and at follow-up. Because Shapiro–Wilk testing showed that all thyroid and migraine outcomes deviated significantly from normality, pre–post comparisons were evaluated with the Wilcoxon signed-rank test, between-group comparisons with the Mann–Whitney U test, and a three-tier non-parametric strategy (Aligned Rank Transform with ART-C contrasts, the Brunner–Langer non-parametric mixed model, and a trimmed-means between-within ANOVA) to analyze time × migraine × gender, adjusted for age and illness duration. Spearman rank correlations with percentile-bootstrap 95% confidence intervals were computed, and both robust MM-regression and rank-based Jaeckel regression were carried out. Another analysis stratified participants by baseline thyroid status: euthyroid vs. subclinical hypothyroidism (SCH). **Results:** After six months of MYSE supplementation, significant reductions were observed in TSH (median 3.60 → 2.80 mIU/L, Wilcoxon *p* < 0.001, rank-biserial r = −0.94), MMDs (14 → 11, *p* < 0.001, r = −0.99), and MSDs (14 → 11, *p* < 0.001, r = −0.99), while fT4 increased slightly (1.30 → 1.50 ng/dL, *p* < 0.001) and fT3 remained stable. For MMAs, a small effect was detected by the paired Wilcoxon test (*p* = 0.002) but the main effect of time did not survive adjustment in any of the three covariate-adjusted mixed models (ART *p* = 0.079; nparLD *p* = 0.55; WRS2 *p* = 0.084). Chronic migraine patients had higher baseline and follow-up headache burden but experienced greater reductions in MMDs. The percentage reduction in TSH was positively correlated with improvement in MMDs (Spearman ρ = 0.45, bootstrap 95% CI 0.31–0.57, *p* < 0.001) and was the only significant predictor in both robust MM-regression (β = 0.28, *p* < 0.001) and rank-based regression (β = 0.25, *p* < 0.001). The TSH–MMD association held within each thyroid-status stratum separately (ρ = 0.42 in euthyroid, ρ = 0.51 in SCH; *p* < 0.001 for both), indicating an individual-level signal rather than a between-group artefact. **Conclusions**: MYSE supplementation was associated with improved thyroid parameters and a meaningful reduction in migraine burden among patients with migraine and HT. The association between TSH reduction and headache improvement supports the hypothesis of an endocrine–metabolic contribution to migraine severity and warrants confirmation in prospective controlled trials. It also supports the clinical value of assessing and addressing thyroid function in this population.

## 1. Introduction

Migraine is a very prevalent form of primary headache characterized by attacks of 4–72-h-lasting, unilateral, throbbing, disabling headache, worsened by movement and associated with nausea, photophobia, and phonophobia [1]. It affects mainly females in their reproductive age and accounts for most of the disability attributed to headache disorders [2]. These characteristics have led several authors to question the biological significance of migraine from an evolutionary perspective, and to consider whether it should be viewed as a disease like any other or as a complex alarm response to excessive allostatic load [3]. Under this point of view, it is worth noting that migraine pathophysiology has several traits that are shared with other pathological conditions that are often comorbid with it [4], suggesting a trigger effect of these conditions on headache attacks. Among these, there is increasing interest in autoimmune thyroiditis [5], especially Hashimoto’s thyroiditis (HT), the leading cause of hypothyroidism, which shares with migraine the high prevalence among females, with a rising prevalence in the general population [6,7]. The presence of HT is suspected in subjects with peculiar clinical picture characterized by symptoms that in part overlap with some ancillary complaints by patients with migraine (for instance, bowel disturbances, sensation of cold, menstrual irregularities, fatigue, muscle pain and cramps, liquid retention, brain fog, memory issues, and depression) and diagnosis of HT depends on detecting circulating antibodies to thyroid antigens, namely thyroperoxidase (TPOAb) and thyroglobulin (TgAb), often associated with reduced echogenicity on thyroid ultrasound [5,8]. Not necessarily does HT lead to hypothyroidism, but if it does, thyroid hormone replacement therapy (THRT) with synthetic thyroid hormones (levothyroxine) can be given [5]. Overt hypothyroidism is diagnosed biochemically when a thyrotropin (TSH) level above the normal range is associated with a low free thyroxine (fT4) level; subclinical hypothyroidism (SCH) occurs when TSH is elevated, but T4 remains within the normal range [9]. In euthyroidism, defined as normal TSH and fT4 levels, selenium supplementation has been suggested to reduce inflammation and TSH levels [10,11], with positive outcomes reported in two recent meta-analyses [8,12,13]. Another food supplement proposed to help with HT is inositol, particularly the isomer myoinositol, which also showed positive laboratory outcomes [14]. When combined, myoinositol and selenium (MYSE) were shown to restore an euthyroid state in Hashimoto’s patients with SCH [15], with a synergistic effect [16] and an echoic pattern improvement [17].

Notably, selenium has already been highlighted as a useful micronutrient supplement for migraine management [18], and the higher is the selenium food intake, the lower is the prevalence of migraine in the general population [19]; on the other hand, inositol is also involved in migraine pathophysiology by a shared genetic background [20] and its brain metabolism is altered both in patients with episodic [21] and chronic [22] migraine (respectively EM and CM).

All these premises provide a strong rationale for hypothesizing that MYSE supplementation could influence migraine in patients with HT, both directly (through micronutrient supplementation) and indirectly (through improved thyroid function).

Since patients with migraine at our outpatient tertiary headache center are referred to an endocrinologist if they test positive for HT and often receive MYSE supplementation, we decided to analyze their clinical records retrospectively to evaluate the effects of the MYSE supplement on their thyroid parameters and headache features.

## 2. Materials and Methods

This is a retrospective cohort study that was conducted at the outpatient clinic of a tertiary headache center, using medical records and headache diary data from migraine patients treated with MYSE supplementation from June 2021 to December 2025. From the diaries, clinical parameters were collected at baseline (after the hormonal assessment but before the start of MYSE supplementation) and at the end of the 6-month treatment period.

The study was conducted in accordance with the STROBE guidelines for observational studies [23] and the Declaration of Helsinki, as revised in 2008.

### 2.1. Participants

Through a manual review of medical records, we identified migraine sufferers who were also diagnosed with HT and had completed at least a 6-month supplementation by MYSE. Inclusion criteria were a diagnosis of migraine according to the International Classification of Headache Disorders, 3rd edition (ICHD-3) criteria [1], having completed and brought the headache diary, structured according to ICHD-3 criteria, to every scheduled follow-up visit, and a positive serology test for HT (TPOAb > 35 IU/mL and TgAb > 40 IU/mL). Some patients already had a diagnosis of HT, although they were not receiving treatment for this condition. In other cases, HT was diagnosed during follow-up, after we added thyroid antibodies and hormone levels to the routine blood tests in the presence of suggestive symptoms, such as intestinal disturbances, feeling cold, menstrual irregularities, fatigue, muscle aches and cramps, water retention, mental fogginess, memory problems, and depression. Moreover, participants were eligible for the study if they had been taking MYSE supplements, prescribed by the endocrinologist at his discretion, for at least 6 months and had undergone thyroid laboratory screening at our hospital laboratory at both the beginning and end of the study. We retrospectively analyzed records of patients who completed the 6-month MYSE treatment period, covering the month before treatment through the last month of treatment by headache diaries, and underwent thyroid hormone rescreening at 6 months: the beginning of the study is defined as the starting date of MYSE assumption and the end as the sixth month of treatment. The screening included TPOAb and TgAb (if not already known), TSH, free triiodothyronine (fT3), and fT4. Exclusion criteria included missing data on baseline or follow-up, changes in THRT, use of preventive treatment for less than 3 months (see Section 2.3 Intervention), or changes to preventive treatment during MYSE supplementation (i.e., patients were either not on prophylaxis or in a stable treatment regimen). Since participants were manually selected retrospectively, there was no need to validate automated codes or algorithms.

### 2.2. MYSE Supplement

For MYSE supplementation, all patients received a commercial fixed-combination product, containing 83 μg of Selenium and 600 mg of myoinositol, whose efficacy in treating thyroid dysfunction has already been documented in the literature [15,16,17,24]. The supplement was prescribed at the endocrinologist’s discretion as part of his consulting activity for patients we referred to him. That specific supplement was chosen because it was the first of its kind on the Italian market and the only one whose use is supported by published studies.

### 2.3. Intervention

The food supplement containing MYSE was taken orally once daily with water, on an empty stomach, for 6 months, regardless of migraine preventive treatments, including beta-blockers, calcium antagonists, tricyclic antidepressants, antiepileptic drugs, and monoclonal antibodies targeting the CGRP molecule or receptor [25]. As noted above, only patients not receiving prophylaxis or that were in a stable preventive treatment were included in the study. The follow-up period lasted 6 months after the start of supplementation. Patient adherence was assessed by anamnestic report at each follow-up visit.

### 2.4. Variables

The use of retrospective, aggregated, and deidentified (for confidentiality) demographic and clinical data from our database in this study was possible because, in compliance with privacy regulations and institutional data use agreement, patients attending our headache center signed a consent form authorizing its use for research purposes (Ethics Committee Lazio 2, n. 0097166/2021, 12 May 2021). All demographic and clinical data (age at onset, migraine duration in years, presence of aura, preventive treatments) from the medical records database used to compile the study were fully available to the investigators. Data cleaning involved identifying and correcting corrupt, inaccurate, or incomplete data through cross-referencing with original medical records. All actions were carefully documented for reproducibility.

#### 2.4.1. Thyroid Parameters

Our hospital laboratory provided thyroid screening results for all parameters (normal ranges: TPOAb ≤ 35 IU/mL; TgAb ≤ 40 IU/mL; TSH 0.4–4.0 mIU/L; fT4 0.7–1.9 ng/dL; fT3 2.0–3.5 pg/mL). According to the presence of HT positivity or hormonal abnormalities, patients were referred for consultation with our endocrinologist consultant. Those who received a medical prescription to treat thyroid dysfunction repeated the hormonal screening after six months of treatment, as part of the standard endocrinological monitoring protocol. They were used to assess the primary outcome of the study.

#### 2.4.2. Headache Parameters

Headache diaries recorded migraine-related hour-by-hour disability on a 1–3 scale (1 = mild disability, all activities allowed without limitation; 2 = moderate disability, most activities limited by about 50%; 3 = severe disability, most activities prohibited), headache duration in hours, and symptomatic medication use. Information on the presence of aura, quality of pain, its location, headache worsening due to routine physical activity, and typical migraine symptoms (photophobia, phonophobia, nausea, vomiting) was also recorded. These measures enabled reliable classification of monthly headache days (MHDs), monthly migraine days (MMDs), monthly migraine attack counts (MMAs; we have regarded as an attack each period of pain preceded and followed by at least 24 h of interictal pain-free period), and monthly symptomatic drug amount (MSDs) according to ICHD-3 criteria [1]. Subjects with MHDs ≥ 15 and at least 8 MMDs were classified as patients with CM; among them, those who used aspirin, non-steroidal anti-inflammatory drugs (NSAIDs) or paracetamol (acetaminophen) for at least 15 days per month, and other acute treatments for at least 10 days per month, were classified as having medication overuse headache (MOH) [1]. Through diary analysis, we determined each patient’s MMDs (at least four hours of headache with typical migraine features, or less if treated with acute medication), MMAs (each episode of headache preceded and followed by a headache-free day), and MSDs used to treat headache, which were used to evaluate the study’s secondary outcome.

### 2.5. Endpoint

The baseline period was defined as the month preceding the initiation of MYSE. The primary endpoint was the change in thyroid hormone parameters to confirm the efficacy of MYSE supplementation also in a population of subjects with migraine. Secondary endpoints included changes in migraine clinical data, namely MMDs, MMAs, and MSDs, following MYSE supplementation.

### 2.6. Statistical Analysis

A priori statistical power calculations were not conducted because the available data determined the sample size. Descriptive statistics were computed for demographic and clinical variables across groups. Continuous variables were summarized as mean ± standard deviation (SD) and as median (first quartile–third quartile, Q1–Q3); the Shapiro–Wilk test was used to assess the normality of each variable at each timepoint. Categorical variables were reported as counts and percentages.

Because the Shapiro–Wilk test indicated that all thyroid (TSH, fT4, fT3) and migraine (MMD, MMA, MSD) outcomes deviated significantly from a normal distribution at both baseline and follow-up (all *p* ≤ 0.003), whereas only illness duration was approximately normal (*p* = 0.068), the originally planned parametric tests were replaced by their non-parametric and robust counterparts. Between-group differences at each timepoint (episodic vs. chronic migraine) were evaluated with the Mann–Whitney U test; effect sizes are reported as the rank-biserial correlation (r_rb) and as the Hodges–Lehmann location shift with 95% confidence interval. Within-subject pre–post changes were evaluated with the Wilcoxon signed-rank test, with the matched-pairs rank-biserial correlation as the effect size; Holm-corrected *p*-values are reported alongside the unadjusted values.

The two originally planned repeated-measures ANCOVAs (time × migraine × gender, adjusted for age and illness duration) were replaced by a three-tier non-parametric strategy. The primary analysis was an Aligned Rank Transform ANOVA (ART) [26] with ART-C contrasts for the time × migraine interaction [27]; age and illness duration were partialled out of each outcome via robust MM-regression prior to alignment and ranking. As a covariate-free confirmation we fitted the Brunner–Langer non-parametric mixed model (nparLD::f2.ld.f1; [28]) and report the ANOVA-type statistic (ATS). As a further robustness check, we fitted a trimmed-means (20%) between-within ANOVA (WRS2::bwtrim; [29]).

Rationale for the multi-method convergent analysis. The originally planned rm-ANCOVA had to accommodate simultaneously a within-subject factor (time), two between-subject factors (migraine chronicity, gender), two continuous covariates (age, illness duration), and non-normal outcomes. No single non-parametric procedure satisfies all four demands at once; each candidate method relaxes different parametric assumptions. ART preserves the full factorial mixed design and relaxes the distributional assumption via alignment-and-ranking, accepting covariates through a two-step residualization procedure. nparLD makes no distributional assumption whatsoever and is fitted without covariates, so it rules out that ART results are driven by the residualization step. WRS2::bwtrim keeps the original interval scale and down-weights the 20% most extreme observations, addressing the outlier-sensitivity component of the normality concern. Convergence across all three methods was therefore interpreted as evidence that the substantive conclusion does not depend on which parametric assumption is relaxed. TSH, fT3, fT4, and MMDs were expressed as percentage change variables, calculated as the proportional difference between baseline and follow-up values. This approach allows comparability across subjects with different baseline levels and reduces scale-related bias.

Bivariate associations between percentage changes in TSH and migraine outcomes were quantified with Spearman’s rank correlation coefficient and percentile-bootstrap 95% confidence intervals (5000 resamples). The multivariate model of MMD percentage change on TSH percentage change, adjusting for fT4%, fT3%, age, illness duration, gender, and migraine type, was re-estimated using both robust MM-regression (robustbase::lmrob) [30], which preserves the interval scale and the clinical interpretation of “% change in MMD per % change in TSH”, and rank-based Jaeckel regression (Rfit::rfit) [31], which makes no distributional assumption; a bootstrap 95% CI for the TSH% coefficient was obtained by resampling rows (5000 replicates). The two methods address two distinct possible objections to OLS (outlier-driven bias and distributional misspecification) and their agreement provides the regression-level analogue of the convergence argument used for the mixed-design analyses.

A pre-planned stratified analysis contrasted baseline euthyroid participants (TSH ≤ 4.0 mIU/L) with those meeting biochemical criteria for subclinical hypothyroidism (TSH > 4.0 mIU/L) on the same non-parametric within- and between-subject tests, and on the within-stratum Spearman correlation between TSH% and MMD%.

All analyses were conducted in R 4.3 [32] with the packages ARTool 0.11.2, nparLD 2.2, WRS2 1.1, rstatix 0.7, effectsize 1.0, robustbase 0.99, Rfit 0.27, DescTools 0.99, and boot 1.3.

Statistical significance was set at *p* < 0.05.

## 3. Results

All data were prospectively collected at the individual patient level, according to our routine clinical practice during the first visit and subsequent follow-up visits, and retrospectively analyzed. The same accredited hospital laboratory measured thyroid hormone levels using standardized assays at baseline and at 6 months of MYSE supplementation, which was prescribed at the endocrinology consultant’s discretion following a multidisciplinary assessment. Headache diaries were maintained prospectively by patients and reviewed at follow-up visits. All the files were manually reviewed by two investigators. Data cleaning involved cross-referencing each entry with the original medical record and headache diary. According to the exclusion criteria, inaccurate or incomplete records were excluded from the analysis. Of the 207 patients who received a MYSE prescription during the study period, 163 (78.7%) met all inclusion criteria and were included in the analysis. The remaining 44 patients (21.3%) were excluded due to missing baseline or follow-up data, or failure to complete the six-month supplementation period.

This retrospective study included 163 adult patients diagnosed with migraine according to ICHD-3 criteria [1], comorbid with HT. At the time of the endocrinologist consultation and MYSE prescription, all subjects were receiving stable migraine preventive treatment in accordance with guidelines [25]; no one was undergoing THRT. The sample comprised 127 women (77.9%) and 36 men (22.1%), with a median age of 46 years (Q1–Q3: 36–53; mean 44.6, SD 12.8). Median disease duration was 26 years (Q1–Q3: 15.5–33; mean 25.3, SD 12.2) and median age at onset was 18 years (Q1–Q3: 13–24; mean 19.3, SD 8.9). Migraine was classified as episodic in 89 participants (54.6%) and chronic in 74 (45.4%); all subjects with CM met criteria for MOH. Fifteen patients had a positive anamnesis for migraine with aura: 12 were in the episodic group, and three were in the CM group. Only 11 of them reported attacks with aura during the MYSE administration period. Full baseline and follow-up descriptive statistics—mean ± SD, median (Q1–Q3) and the Shapiro–Wilk *p*-value for every continuous variable in the whole cohort and by migraine group—are provided in Table 1.

**Figure 1:**
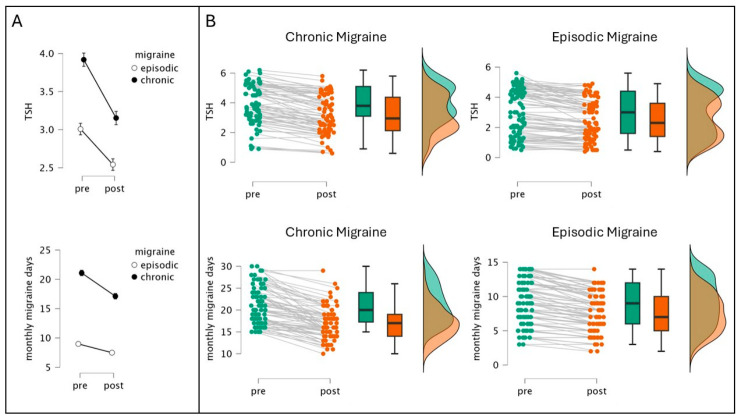
Changes in TSH levels and monthly migraine days following treatment in chronic and episodic migraine patients. (**A**) Mean changes in TSH (top panel) and monthly migraine days (bottom panel) from pre- to post-treatment timepoints, shown separately for episodic (open circles) and chronic (filled circles) migraine patients. Error bars represent the 95% confidence intervals. (**B**) Individual patient trajectories (gray lines connecting green/teal pre-treatment to orange post-treatment values), boxplots showing median and interquartile ranges, and density distributions for TSH levels (top panels) and monthly migraine days (bottom panels) in chronic migraine (left) and episodic migraine (right) patients.

At baseline (Figure 1A, left column), patients with CM exhibited a markedly higher headache burden compared to patients with EM: MMDs, 21.1 vs. 9.0 (*p* < 0.001); MMAs, 7.4 vs. 5.2 (*p* < 0.001); and MSDs, 22.4 vs. 9.5 (*p* < 0.001). MMDs median 20 vs. 9 (Mann–Whitney W = 0, *p* < 0.001, r_rb = −1.00, Hodges–Lehmann shift −12 days); MMAs 7 vs. 5 (W = 1644, *p* < 0.001, r_rb = −0.50); MSDs 21 vs. 10 (W = 70.5, *p* < 0.001, r_rb = −0.98). Endocrine differences were also observed: TSH levels were higher in CM patients (3.80 vs. 3.00 mIU/L; W = 2177.5, *p* < 0.001, r_rb = −0.34); however, fT4 and fT3 did not differ significantly between groups.

Wilcoxon signed-rank tests revealed significant pre-to-post changes in most thyroid and migraine parameters. TSH decreased from a median of 3.60 to 2.80 mIU/L (V = 402.5, *p* < 0.001, r_rb = −0.94, HL median difference = −0.55 mIU/L); fT4 increased from 1.30 to 1.50 ng/dL (V = 13,366, *p* < 0.001, r_rb = +1.00); and fT3 was stable (2.80 to 2.70, *p* = 0.72). MMDs fell from 14 to 11 (V = 72, *p* < 0.001, r_rb = −0.99, HL = −2.5 days) and MSDs from 14 to 11 (V = 34, *p* < 0.001, r_rb = −0.99, HL = −3). Monthly migraine attacks showed a small reduction detectable by the paired signed-rank test (median unchanged at 6; V = 948, *p* = 0.002, r_rb = −0.37, Holm-adjusted *p* = 0.004), a finding qualified further by the covariate-adjusted mixed-design analyses below. All Holm-adjusted *p*-values for significant results remained < 0.001 except for MMA (*p* = 0.004).

Mann–Whitney tests revealed differences in post-treatment headache severity between migraine groups (Figure 1A, right column). Despite overall improvement, CM patients maintained a higher headache burden (MMDs median 17 vs. 7, *p* < 0.001; MSDs 17 vs. 7, *p* < 0.001) and higher TSH levels (2.95 vs. 2.30 mIU/L, *p* = 0.004). Figure 1B depicts the changes of TSH and MMDs at the individual level in CM and EM.

### 3.1. Non-Parametric Mixed-Design Analyses

Table S1 (Supplementary Materials) summarizes non-parametric mixed-design results for TSH, MMD, and MSD, reporting the effect statistic (F, ATS, or Q) and *p*-value for time, migraine, gender, and their interactions under ART, nparLD, and WRS2::bwtrim.

Three complementary models were fitted for each outcome, all converging on the same pattern for the two primary variables (Table S1). For TSH, ART revealed a significant main effect of time (F = 133.4, *p* < 0.001), of migraine (F = 7.39, *p* = 0.007) and a time × migraine interaction (F = 6.92, *p* = 0.009); these results were confirmed by nparLD (Time ATS = 125.3, *p* < 0.001; GroupA × Time ATS = 4.39, *p* = 0.036) and by WRS2::bwtrim (Q_time = 162.7, *p* < 0.001; Q_migraine × time = 9.45, *p* = 0.003). No three-way interaction involving gender reached significance across methods. For MMD, all three methods showed very strong time and migraine main effects and a robust time × migraine interaction (ART F = 37.8, *p* < 0.001; nparLD GroupA × Time ATS *p* < 0.001; WRS2 Q_migraine × time = 23.9, *p* < 0.001). For MSD the same pattern held (time × migraine *p* < 10^−8^ across methods). fT4 showed a very strong main effect of time (ART F = 628, *p* < 0.001) with no reliable interactions. fT3 showed no significant effects in any method. For MMA, the main effect of time did not reach significance in any of the three covariate-adjusted mixed models (ART F = 3.12, *p* = 0.079; nparLD Time ATS *p* = 0.55; WRS2 *p* = 0.084), indicating that the small signed-rank effect reported above does not survive adjustment for age, illness duration, gender, and the group structure.

**Figure 2:**
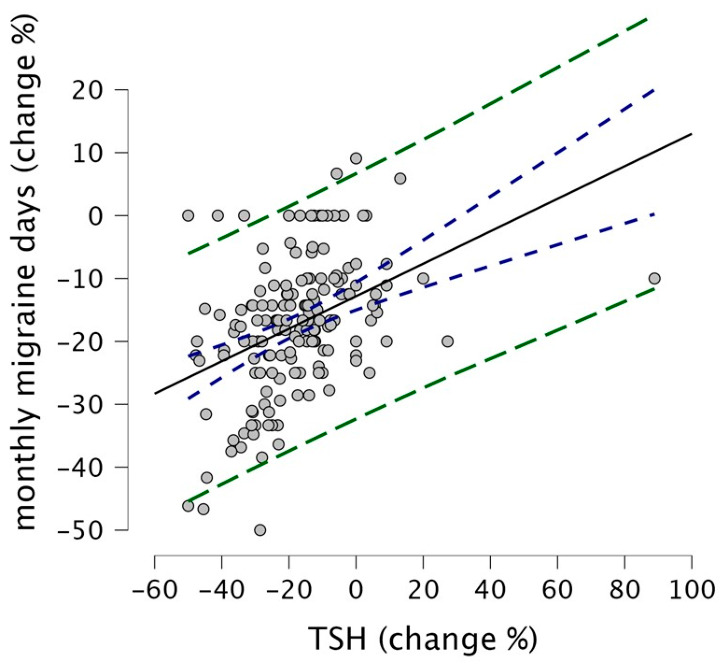
Correlation between percentage changes in TSH levels and monthly migraine days. Scatter plot showing the relationship between TSH change (%) and monthly migraine days change (%) across all patients. The solid black line represents the regression line, blue dashed lines indicate 95% confidence intervals, and green dashed lines show 95% prediction intervals. Each point represents an individual patient.

Correlation analyses (Figure 2) revealed that the percentage of reduction in TSH levels was positively correlated with fewer MMDs (Spearman ρ = 0.45, percentile bootstrap 95% CI 0.31–0.57, *p* < 0.001). No significant associations were found between improvement in migraine and age or disease duration (Spearman ρ = −0.05, *p* = 0.49 for age; ρ = −0.07, *p* = 0.35 for illness duration). Changes in fT4 and fT3 were not correlated with clinical outcomes (ρ = −0.08, *p* = 0.29; ρ = 0.00, *p* = 0.99, respectively).

The robust MM-regression model, including gender, chronicity, age, disease duration, and thyroid hormone changes, accounted for 18.3% of the variance in MMD improvement; TSH% was the only significant predictor (β = 0.28, SE = 0.052, t = 5.35, *p* < 0.001; bootstrap 95% CI 0.15–0.47). The rank-based regression (Rfit::rfit) produced concordant estimates (β_TSH% = 0.25, *p* < 0.001). Other variables, including changes in fT4 and fT3, were not statistically significant in either model.

### 3.2. Stratified Analysis by Baseline Thyroid Status 

A pre-planned analysis stratified participants by baseline TSH: 96 were classified as euthyroid (TSH ≤ 4.0 mIU/L) and 67 as having subclinical hypothyroidism (SCH; TSH > 4.0). Both subgroups showed significant reductions in TSH, MMD, and MSD at 6 months (all *p* < 10^−11^). MMA decreased significantly only in the SCH subgroup (V = 27, *p* < 0.001) and not in euthyroid (*p* = 0.50). The absolute reduction in TSH was larger in the SCH group (Hodges–Lehmann shift 0.30 mIU/L, *p* = 0.001) but the reduction in MMD was comparable across strata (*p* = 0.62). At follow-up, 29/67 SCH patients (43.3%) had reached biochemical euthyroidism, whereas 95/96 euthyroid patients (98.9%) remained euthyroid. Crucially, the TSH%–MMD% correlation held within each stratum separately: ρ = 0.42 (95% CI 0.23–0.58, *p* < 0.001) in euthyroid and ρ = 0.51 (95% CI 0.27–0.69, *p* < 0.001) in SCH, indicating that the whole-cohort correlation reflects an individual-level signal rather than a between-group artefact.

## 4. Discussion

The first observation from our retrospective analysis of data from a cohort of 163 subjects with migraine and HT was that 6 months of MYSE supplementation was associated with a significant reduction in TSH (Hodges–Lehmann median difference = −0.55 mIU/L, rank-biserial r = −0.94), consistent with prior literature [33]. The magnitude of the TSH reduction is independent of the “time × migraine × gender” interaction, is unaffected by age- and illness-duration-adjustment, and is preserved across all three mixed-design methods. This result suggests that MYSE modulates thyroid function in this peculiar population as well. It is not surprising, since the same MYSE supplement has already been shown to be effective in reducing TSH in subjects with SCH, with and without HT, and in HT patients with TSH values in the higher part of the normal range (>2.5 mIU/L) [14,15,16,17,24,33,34,35]. This result could be due to the combined effect of selenium and myoinositol. Selenium is an essential trace element because the enzymes that depend on it perform critical antioxidant and anti-inflammatory functions [36]. These enzymes have a significant impact on the immune system and are linked to reduced inflammation in patients with autoimmune thyroiditis. Glutathione peroxidase, for example, is a selenium-dependent enzyme that can neutralize hydrogen peroxide and lipid and phospholipid hydroperoxides [36]. This action limits the spread of reactive oxygen species and free radicals, reducing the synthesis of critical proinflammatory molecules, such as prostaglandins and leukotrienes [37]. Finally, a selenium-dependent enzyme, iodothyronine deiodinase, catalyzes the conversion of the precursor fT4 to the more biologically active thyroid hormone fT3 [36]. On the other hand, myoinositol acts as a second messenger, modulating the activity of hormones such as TSH, follicle-stimulating hormone (FSH), and insulin. In the context of the TSH signaling cascade, myoinositol also regulates hydrogen peroxide-mediated iodination, a critical step in thyroid hormone synthesis [16]. Thus, our results further confirm the influence of MYSE supplementation on thyroid function.

The second result we observed in our study, based on monitoring migraine clinical features (MMDs, MMAs, and MSDs), was a decrease in MMDs and MSDs; despite a similar number of attacks, these attacks were shorter and/or more responsive to acute treatments. These improvements appear to correlate with TSH levels: the greater the reduction in TSH, the greater the improvement in these measures. The Spearman rank correlation between TSH% and MMD% holds within both baseline thyroid subgroups separately (ρ = 0.42 in euthyroid, ρ = 0.51 in SCH), which is an important argument against the interpretation that the correlation is merely a between-group artefact reflecting different baseline TSH levels in EM and CM. Interestingly, at baseline, TSH values in the CM group were higher than those in the other group, suggesting a role for this hormone as a predictor of disease severity; this suggestion is consistent with the results of the multivariate robust and rank based regression models, in which the only significant predictor of MMD improvement was the decrease in TSH, while the model excluded fT4 or fT3 increase, gender, chronicity, patients’ age, and disease duration. Notably, the reduction in monthly migraine attack frequency was detectable in the unadjusted signed-rank test but did not survive adjustment in any of the three covariate-adjusted mixed-design models: the number of attacks did not change with the treatment, suggesting that the supplementation does not prevent attack recurrence but may shorten their duration, increase responsiveness to acute treatment, or both. The reduction in the need for acute therapies may reflect a MYSE-induced shorter attack duration (fewer days with headache at the same attack frequency) or an improvement in treatment response. Interestingly, the higher reduction in terms of MMD was observed in the CM group, maybe because they started from a higher headache frequency (and TSH level); however, at the end of the six months of supplementation, although more reduced than in the episodic group, the burden of their migraine remains higher than that of the other group (meanly, they start chronic and end chronic).

Overall, our results support previous observations of a possible link between the degree of thyroid dysfunction and the severity of migraine symptoms [38,39,40], suggesting a beneficial secondary effect of the integrative treatment on migraine.

These findings support our hypothesis that combined MYSE supplementation benefits individuals with migraine and HT. The concurrent reduction in TSH levels and migraine attack frequency suggests a possible synergistic effect between the two compounds.

A notable addition from the stratified analysis is that 43.3% (29/67) of SCH patients reached biochemical euthyroidism at 6 months, whereas 98.9% of baseline-euthyroid patients remained euthyroid. This is consistent with the known clinical effect of MYSE in SCH [11,12] and provides a clinically meaningful endpoint beyond the pooled TSH reduction.

**Figure 3:**
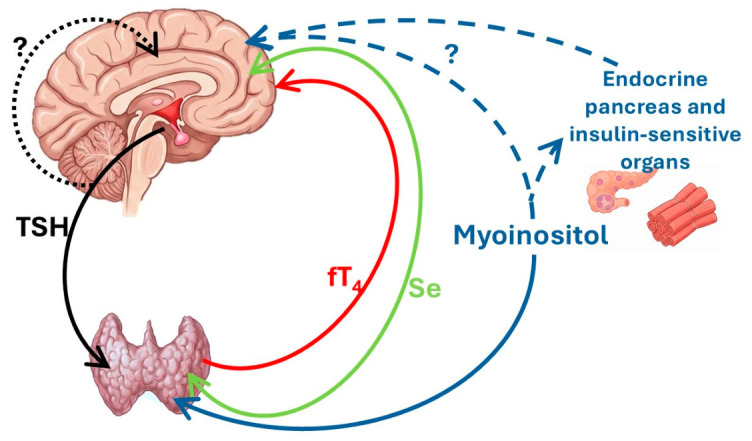
Proposed hypothetical model of the mechanisms linking thyroid–metabolic modulation by MYSE supplementation to migraine improvement. This figure is schematic and speculative; it is not based on direct experimental evidence from the present study. Black arrows depict the thyrotropin (TSH) action from the hypothalamus/pituitary to the thyroid gland; the dashed line with the question mark (?) indicates a putative direct effect of TSH on the brain. The red arrow indicates the role of thyroxine (fT4) in the brain. The green arrow indicates the dual supportive role of selenium (Se) in the thyroid and the brain. The blue arrows indicate myoinositol’s modulatory pathway that enhances TSH signaling; the blue dashed arrows with the question mark (?) indicate a putative direct effect of myoinositol on the brain and an indirect impact via insulin modulation, which may protect the brain from migraine by tapering the insulin resistance.

These results can be interpreted in light of some pathophysiological hypotheses (Figure 3).

### 4.1. Effect of Thyroid Hormones on Migraine

The relationship between hypothyroidism and migraine has been well known for many years [38]; thus, it is possible to speculate that reversion to euthyroidism can lead to migraine improvement. Elevated fT4 levels and normalized TSH may boost neuronal metabolism and prevent migraine. Previous research has shown that THRT can decrease the frequency and severity of migraine attacks in hypothyroid patients, probably by improving cerebral metabolic balance and altering pro-inflammatory neuropeptides, both in adult [41,42,43] and pediatric patients [44]; moreover, compared to adherent ones, low adherence to levothyroxine treatment in people with hypothyroidism is related to more likely to be comorbid with migraine [45]. However, none of our patients were under THRT during the observation period, and fT4 levels did not appear to be responsible for the observed changes in migraine parameters.

### 4.2. Role of Selenium in Migraine

A higher risk of developing migraine was observed in the presence of selenium-deficient dietary intake, regardless of the thyroidal hormone status [19,46,47]. The most prevalent hypothesis to explain this phenomenon calls into question the pathophysiological migraine mechanism of higher oxidative stress [48] that can be tapered by selenium [47]. Through its antioxidant activity and modulation of selenoproteins, selenium may help reduce oxidative stress and systemic inflammation. This mechanism could reduce susceptibility to migraine attacks. In fact, clinical studies have shown that selenium supplementation improves the frequency and intensity of migraine attacks and reduces markers of oxidative stress in patients with migraine, suggesting an optimal daily dose of 93 μg [18], very close to the amount present in the MYSE supplement (83 μg) prescribed to our patients.

### 4.3. The Putative Role of Myoinositol as a Metabolic and Glial Mediator in Migraine

To the best of our knowledge, this is the first time myoinositol supplementation has been discussed in the context of migraine management. It warrants detailed discussion, including the complex role of this carbocyclic polyalcohol in humans. Myoinositol is an isomer of inositol, and about 4 g are endogenously produced from glucose-6-phosphate every day [49], mainly in the kidneys, but also in other tissues such as the brain and reproductive organs [50]. Additionally, it is absorbed through the diet [49], at approximately 1 g per day, with primary sources including cereals, legumes, oilseeds, and nuts [50]. However, due to the widespread and increasing consumption of highly processed foods, daily myoinositol absorption is decreasing [49]. In addition, alterations in the gut microbiota may further reduce daily absorption, thereby inducing myoinositol deficiency [51]. Myoinositol functions as an osmolyte, as a precursor to intracellular second messengers (inositol triphosphate, which transmits several endocrine signals including FSH, TSH, and insulin), and as a component of structural lipids like phosphatidyl-inositol and its various phosphate lipid derivatives [52]; moreover, it has been shown to have a vital antioxidant effect [53,54,55], further characterizing this molecule as a multifunctional compound essential for life. As discussed above, migraine pathophysiology involves oxidative stress [48] and thyroid dysfunction [6,20,39,40,56]; in addition, it is associated with insulin resistance [57,58] and polycystic ovary syndrome [59], both of which are currently treated with myoinositol supplementation. Because of its effects on insulin sensitivity and thyroid metabolism, myoinositol, aside from its role in oxidative stress, could be regarded as a primary driver of human energy balance and thereby help prevent the energy deficits commonly observed in migraine sufferers [60]. Moreover, as reported above, there is a genetic correlation between migraine and thyroid dysfunction that is mediated by the inositol pathway. One shared locus includes the ITPK1 (inositol 1,3,4-trisphosphate 5/6-kinase) gene, which directly regulates inositol metabolism and the production of inositol phosphate second messengers [20]. Furthermore, two genes involved in inositol phosphate-mediated signaling, PLCE1 (phospholipase C epsilon 1) and NMUR2 (neuromedin U receptor 2), were reported to be associated with migraine susceptibility in a genome-wide association study [61]. Furthermore, the INPP5D (inositol polyphosphate-5-phosphatase D) gene is involved in NLRP3 inflammasome activation [62], which, in turn, is considered a key player in migraine pathophysiology due to its role in pain sensitivity and inflammation [63].

Finally, myoinositol is also regarded as a marker of glial activation; in particular, its diffusion on magnetic resonance imaging (MRI) spectroscopy closely reflects astrocytic morphology and hypertrophy secondary to activation [64]. Spectroscopic MRI studies revealed that patients with episodic and chronic migraine have several alterations of myoinositol levels in different brain structures [21,22,65,66] and within white matter lesions [67], with a positive association with thalamic myoinositol concentration and migraine severity in terms of disease duration and frequency of attacks [68]. In contrast, a negative association was observed between the myoinositol laterality index in the anterior cingulate cortex and the number of days per month of acute medication use [22]. On the other hand, it was observed that in patients with migraine comorbid with major depressive disorder, there were increased myoinositol levels within the dorsolateral prefrontal cortex, suggesting a role for this molecule at the basis of the co-occurrence of these diseases [69]. However, even if myoinositol supplementation could have a putative effect on improving hormonal dysfunctions, inflammation, oxidative stress, and energy metabolism in the patients in the present study, it is unclear whether it can also directly act on brain inositol-mediated dysfunctions. On the one hand, myoinositol is hydrophilic and unable to cross the blood–brain barrier (BBB) to enter the central nervous system (CNS) at doses below 12 g per day [70]. On the other hand, there are three different myoinositol transporters, namely sodium/myoinositol transporter-1 (SMIT1), sodium/myoinositol transporter-2 (SMIT2), and H^+^/myoinositol transporter (HMIT), that are also expressed in the brain [52] and can provide this transport at lower concentrations. In addition, some CNS sites are outside the BBB (e.g., subarachnoid space/pial surface, circumventricular organs) [71] and can be influenced by myoinositol supplementation.

Complementarily, somehow, inositol is also involved in modulating preventive treatment. In fact, DL-propranolol administration (a drug used in migraine prophylaxis) increases, in a time- and concentration-dependent manner, the production of inositol phosphate [72]. Additionally, single-nucleotide polymorphisms in genes involved in myo-inositol biosynthesis can predict the efficacy of verapamil as preventive treatment in migraine and cluster headache [73].

### 4.4. A Possible Role for TSH in Migraine

If higher levels of TSH are related to a worse headache picture, and its reduction to higher MMD tapering, it is possible to speculate about a potential direct negative influence of this hormone on migraine. Indeed, TSH receptors are also expressed in various regions of the CNS, including: amygdala, cerebellum, cingulate gyrus, frontal lobe/cortex, hippocampus, hypothalamus, occipital lobe, parietal cortex, parieto-occipito-temporal cortex, primary motor cortex, primary sensory cortex, pituitary gland, thalamus, and temporal lobe [47]. Still, their role remains unclear, but they were supposed to be related to Alzheimer’s disease, bipolar disorder [74], and feeding control [75]. Thus, based on our results, we cannot dismiss their potential role also in the pathophysiology of migraine.

Alternatively, we can view TSH as a proxy for metabolic status, with higher levels observed in micronutrient deficiencies, of selenium and myoinositol. Blood tests can detect the first deficiency, but it is not routinely performed; the second cannot be detected in clinical practice. Supporting this idea, we can regard the observed results in our patients in an alternative way: MYSE is not a simple cure for HT or hypothyroidism (it can also lower TSH levels even in euthyroid or hypothyroid without HT individuals [35]); instead, it may serve as a nutrient replenishment for the whole body that is deficient in these micronutrients. The thyroid may be the first organ to provide an early sign of this shortening, with hypoactivity that induces a secondary increase in TSH. Thus, reduced availability of myoinositol or selenium can raise TSH levels, indicating increased nutritional requirements that may exacerbate migraine, which may, in turn, benefit from MYSE supplementation. From this perspective, the migraine improvement observed in patients receiving levothyroxine therapy [41,42,43] may be interpreted as an indirect effect of this mechanism. Hormonal replacement therapy suppresses endogenous thyroid T4 production through negative feedback on TSH, thereby reducing the thyroid’s demand for myoinositol and selenium. As a result, these micronutrients may become more available for other tissues and metabolic pathways, potentially contributing to migraine improvement.

### 4.5. Clinical Implications

These findings support the hypothesis that thyroid function, monitored by TSH levels, may influence migraine severity and treatment response and should be detected in CM patients and in the presence of clinical symptoms suggestive of hypothyroidism. Checking TSH levels can help identify patients who are more likely to benefit from specific interventions. Importantly, the stratified analysis shows that the TSH–migraine link is present in both patients with SCH and those with baseline TSH within the reference range, suggesting that the clinical benefit of MYSE is not confined to patients with overt thyroid dysfunction. Further research should investigate whether TSH modulation, despite HT, is a direct therapeutic target or an indirect marker of systemic changes associated with migraine improvement. For instance, in the presence of euthyroidism in HT patients, MYSE can reduce TSH; it may also be the same for patients with migraine without HT, but with TSH values in the higher part of the normal range (>2.5 mIU/L).

## 5. Limitations

While our findings are significant and consistent with prior research, we acknowledge that certain methodological shortcomings may have influenced our results, limiting their generalizability.

First, this is a retrospective observational study; given the design, we cannot assess whether MYSE supplementation improves migraine outcomes, as potential confounding factors or information bias could have influenced our observations.

Second, because all patients were on stable preventive treatments, we cannot generalize the findings to untreated populations or exclude additive or synergistic effects of ongoing prophylaxis.

Third, there is no comparator group receiving selenium alone, myoinositol alone, or placebo; therefore, we cannot determine whether the beneficial effect in migraine is attributable solely to selenium supplementation [18], whether myoinositol plays an individual role in the observed outcome, or whether, as previously reported for HT, there is synergy between the components.

Fourth, although the stratified analysis contrasting baseline euthyroid and subclinical hypothyroid participants provides a first answer to the question of whether MYSE supplementation should be limited to patients with HT within a specific TSH range, in the study we did not identify a TSH threshold leaving open the question of whether MYSE supplementation should be limited to patients with HT within a specific TSH range or considered regardless of thyroid status.

Fifth, individual dietary intake data for selenium and myoinositol were not collected in our retrospective database and cannot be reconstructed from medical records.

Sixth, the heterogeneity of preventive migraine treatments across our cohort—encompassing multiple drug classes and a substantial proportion of patients on combination regimens—precluded stratification by prophylactic therapy. Patients on polytherapy cannot be reliably assigned to a single-drug subgroup, and the resulting sub-samples would be too small for meaningful statistical inference. The type of preventive treatment, therefore, remains an uncontrolled variable in our study, and future prospective studies should incorporate the prophylactic regimen as a stratification factor at the design stage or as an exclusion criterion.

Seventh, TSH is subject to physiological variations according to the seasonal variation, body weight changes, and spontaneous TSH normalization in subjects with SCH, which may affect serial measurements. Since each patient served as their own control in the within-subject comparisons and the recruitment spanned all calendar months symmetrically across the baseline and subsequent six-month assessments, it is not possible for a systematic seasonal bias acting uniformly in the same direction to account for the observed median TSH reduction: the observed magnitude is substantially in line with the seasonal swings reported in SCH populations if all subjects were recruited in the winter and retested in the summer [76], but this has not occurred. Further, spontaneous TSH normalization occurs in approximately 40% of subjects with SCH [77], and cannot be excluded as a partial contributor to the observed migraine benefit; this concern is mitigated by the stratified analysis: the comparable reduction in migraine day frequency observed in baseline-euthyroid patients—in whom no SCH was present, to spontaneously remit—argues against spontaneous normalization as the sole explanatory mechanism. Furthermore, the within-stratum TSH%–MMD% correlation was reproduced in both subgroups, including the euthyroid one. Unfortunately, body weight data were not systematically recorded in the clinical files, precluding covariate adjustment for this dynamic confounder; this represents a genuine limitation inherent to the retrospective design. Finally, although the multi-method convergent analytical strategy—encompassing ART, nparLD, WRS2::bwtrim, MM-regression, and rank-based regression—provides robust evidence that the substantive findings do not depend on any single parametric assumption, convergence arguments are informative only insofar as the candidate methods relax distinct assumptions. The within-stratum correlation analysis, which relies on no mixed model and nonetheless reproduces the TSH–MMD association, partially mitigates this concern. Taken together, these considerations confirm that the present findings remain associative. A pre-registered prospective trial—incorporating serial TSH measurements stratified by season and body-weight trajectory—will be required to establish causal inference.

Finally, the retrospective nature of our study implies the risk of selection, information, and completion bias. Nonetheless, selection bias was minimized by applying pre-specified inclusion and exclusion criteria uniformly to all available records from consecutive patients; no convenience or voluntary sampling was involved. On the other hand, information bias was limited by the following precautions: thyroid parameters were measured at a single accredited hospital laboratory using standardized assays; headache data were extracted from structured headache diaries using ICHD-3 criteria, thereby reducing the risk of differential misclassification. However, we acknowledge the risk of residual information bias. Also, completion bias cannot be excluded since patients who discontinued treatment or were lost to follow-up may differ systematically from those included, potentially leading to an overestimation of adherence-related benefits. Together, these limitations, along with the lack of evaluation of higher myoinositol doses that could more reliably cross the blood–brain barrier, limit conclusions about the underlying neurobiological mechanisms and the long-term sustainability and generalizability of the effects.

Future randomized controlled trials testing different dosages and combinations of selenium and myoinositol over more extended follow-up periods are warranted to confirm these findings and better define which subgroups of patients with migraine and HT may derive the greatest benefit. Additionally, Future prospective studies should incorporate validated food frequency questionnaires to account for dietary exposure. Finally, the metabolic role of inositol in glial homeostasis and neuronal metabolism in the CNS deserves further exploration.

## 6. Conclusions

The results of this retrospective uncontrolled cohort study suggest that supplementing with a combination of MYSE in patients with HT who also experience episodic or chronic migraine influences thyroid function, resulting in a significant reduction in TSH levels and meaningful reductions in headache days and analgesic use. Patients with CM exhibited more severe initial and final profiles, although they derived benefit from the treatment. The lack of significant changes in attack frequency suggests that the intervention mainly affected headache duration rather than resilience to triggers.

The strong association between TSH reduction and headache improvement suggests an endocrine role in migraine pathophysiology. While fT4 increased slightly and fT3 remained stable, these changes did not align with migraine clinical outcomes, suggesting that TSH may be a more relevant biomarker and supporting its role as a metabolic proxy.

These findings underscore the importance of recognizing the association between HT and migraine, as improving thyroid health can improve headache management.

In the absence of a control group, causal inference is not possible in this retrospective, uncontrolled cohort study; the observed changes may, in part, reflect spontaneous disease fluctuations, regression to the mean, or other unmeasured factors. These results should be interpreted as hypothesis-generating, warranting confirmation in randomized placebo-controlled trials.

## Figures and Tables

**Table 1:** Descriptive statistics—whole cohort and by migraine group.

Variable	Whole Cohort (*n* = 163)	EM (*n* = 89)	CM (*n* = 74)
Age (years)	44.6 ± 12.8|46 (36–53) (*p* < 0.001)	44.9 ± 12.2|46 (36–54) (*p* = 0.042)	44.2 ± 13.5|48.5 (36.3–52.8) (*p* < 0.001)
Age at onset (years)	19.3 ± 8.9|18 (13–24) (*p* < 0.001)	19.8 ± 9.0|18 (14–25) (*p* = 0.006)	18.6 ± 8.8|17 (12.3–23) (*p* = 0.003)
Illness duration (years)	25.3 ± 12.2|26 (15.5–33) (*p* = 0.068)	25.1 ± 12.0|26 (16–34) (*p* = 0.333)	25.6 ± 12.5|25.5 (15.5–32.8) (*p* = 0.074)
TSH pre (mIU/L)	3.42 ± 1.50|3.60 (2.15–4.70) (*p* < 0.001)	3.01 ± 1.53|3.00 (1.60–4.40) (*p* < 0.001)	3.92 ± 1.32|3.80 (3.10–5.10) (*p* = 0.029)
TSH post (mIU/L)	2.82 ± 1.34|2.80 (1.80–3.95) (*p* < 0.001)	2.54 ± 1.32|2.30 (1.40–3.60) (*p* < 0.001)	3.15 ± 1.30|2.95 (2.12–4.38) (*p* = 0.036)
fT4 pre (ng/dL)	1.26 ± 0.42|1.30 (0.90–1.60) (*p* < 0.001)	1.29 ± 0.42|1.40 (1.00–1.60) (*p* = 0.012)	1.22 ± 0.43|1.20 (0.90–1.58) (*p* = 0.006)
fT4 post (ng/dL)	1.41 ± 0.46|1.50 (1.00–1.80) (*p* < 0.001)	1.44 ± 0.46|1.60 (1.10–1.80) (*p* = 0.003)	1.37 ± 0.47|1.30 (1.00–1.78) (*p* = 0.002)
fT3 pre (pg/mL)	2.76 ± 0.59|2.80 (2.20–3.20) (*p* < 0.001)	2.82 ± 0.55|2.80 (2.30–3.30) (*p* = 0.002)	2.68 ± 0.64|2.70 (2.10–3.18) (*p* = 0.003)
fT3 post (pg/mL)	2.77 ± 0.64|2.70 (2.30–3.20) (*p* = 0.044)	2.86 ± 0.64|2.80 (2.40–3.20) (*p* = 0.316)	2.66 ± 0.62|2.55 (2.20–3.10) (*p* = 0.082)
MMD pre	14.5 ± 7.1|14 (8.5–20) (*p* < 0.001)	8.99 ± 3.24|9 (6–12) (*p* = 0.001)	21.1 ± 4.3|20 (17.3–24) (*p* = 0.002)
MMD post	11.9 ± 5.8|11 (7–16) (*p* < 0.001)	7.49 ± 2.80|7 (5–10) (*p* = 0.023)	17.1 ± 3.7|17 (14–19) (*p* = 0.079)
MMA pre	6.20 ± 2.74|6 (4–8) (*p* = 0.003)	5.21 ± 2.46|5 (3–6) (*p* < 0.001)	7.38 ± 2.61|7 (6–9) (*p* < 0.001)
MMA post	6.05 ± 2.46|6 (4–8) (*p* = 0.001)	4.91 ± 2.24|5 (3–6) (*p* < 0.001)	7.42 ± 1.97|7 (6–9) (*p* = 0.004)
MSD pre	15.4 ± 7.8|14 (9–21) (*p* < 0.001)	9.53 ± 3.56|10 (7–12) (*p* = 0.028)	22.4 ± 5.2|21 (20–24) (*p* < 0.001)
MSD post	11.9 ± 6.1|11 (7–16) (*p* < 0.001)	7.55 ± 2.81|7 (5–9) (*p* = 0.021)	17.2 ± 4.6|17 (14–20) (*p* = 0.049)

All continuous variables are reported as mean ± SD and median (Q1–Q3). SW *p* = Shapiro–Wilk *p*-value for normality at that timepoint; *p* < 0.05 rejects normality. EM, episodic migraine; CM, chronic migraine; TSH, thyroid-stimulating hormone; fT4, free thyroxine; fT3, free triiodothyronine; MMD, monthly migraine days; MMA, monthly migraine attacks; MSD, monthly symptomatic drug amount.

## Data Availability

The data presented in this study are available on request from the corresponding author. The data are not publicly available due to privacy and ethical restrictions.

## Supplementary Materials

The following supporting information can be downloaded at: https://www.mdpi.com/article/10.3390/nu18101554/s1, Table S1. Primary non-parametric mixed-design analyses for TSH and MMD.

## Abbreviations

The following abbreviations are used in this manuscript:

MYSE	Myoinositol and Selenium
HT	Hashimoto’s thyroiditis
TSH	Thyrotropin
fT4	free Thyroxine
fT3	free Triiodothyronine
MMDs	Monthly Migraine Days
MMAs	Monthly Migraine Attacks
MSDs	Monthly Symptomatic Drug use
TPOAb	Antibodies anti-Thyroperoxidase
TgAb	Antibodies anti-Thyroglobulin
THRT	Thyroid Hormone Replacement Therapy
EM	Episodic Migraine
CM	Chronic migraine
ICHD-3	International Classification of Headache Disorders, 3rd edition
MHDs	Monthly Headache Days
NSAIDs	Non-Steroidal Anti-Inflammatory Drugs
MOH	Medication Overuse Headache
SD	Standard Deviation
χ^2^	Chi-square
rm-ANCOVA	repeated-measures Analysis of Covariance
DV	Dependent Variable
IV	Independent Variable
FSH	Follicle-Stimulating Hormone
MRI	Magnetic Resonance Imaging
BBB	Blood–Brain Barrier
CNS	Central Nervous System
SMIT1	Sodium/Myoinositol Transporter-1
SMIT2	Sodium/Myoinositol Transporter-2
HMIT	H^+^/myoinositol transporter
