# Supplementary material for: Myoinositol and Selenium (MYSE) Supplementation Is Associated with Favorable Changes in Thyroid Parameters and Migraine Outcomes in Patients with Migraine and Hashimoto’s Thyroiditis: A Retrospective Cohort Study
Source: Nutrients. 2026 May 14;18(10):1554. doi: 10.3390/nu18101554 (PMC13210118; doi:10.3390/nu18101554)
Supplement: Supplementary file 1 [file nutrients-18-01554-s001.zip › nutrients-4222118-supplementary.pdf]

Table S1. Primary non-parametric mixed-design analyses for TSH and MMD

All three methods were fitted on the three-way mixed design (time × migraine × gender). Covariates age and illness duration were partialled out of the ART model only (nparLD and WRS2 do not accommodate continuous covariates, so they serve as assumption-free confirmations).

Outcome	Method	Effect	Statistic (df)	p
TSH	ART	time	F = 133.4 (1, 159)	< .001
TSH	ART	migraine	F = 7.39 (1, 159)	0.007
TSH	ART	time × migraine	F = 6.92 (1, 159)	0.009
TSH	nparLD (ATS)	time	125.3 (1)	< .001
TSH	nparLD (ATS)	migraine × time	4.39 (1)	0.036
TSH	WRS2::bwtrim	time	Q = 162.7 (1, 105)	< .001
TSH	WRS2::bwtrim	migraine × time	Q = 9.45 (1, 105)	0.003
MMD	ART	time	F = 173.5 (1, 159)	< .001
MMD	ART	migraine	F = 315.1 (1, 159)	< .001
MMD	ART	time × migraine	F = 37.8 (1, 159)	< .001
MMD	nparLD (ATS)	time	149.6 (1)	< .001
MMD	nparLD (ATS)	migraine × time	12.2 (1)	< .001
MMD	WRS2::bwtrim	time	Q = 129.5 (1, 63)	< .001
MMD	WRS2::bwtrim	migraine × time	Q = 23.9 (1, 63)	< .001
